# Administration of *Lactobacillus plantarum* GUANKE alleviates SARS-CoV-2-induced pneumonia in mice

**DOI:** 10.1128/spectrum.01603-24

**Published:** 2024-10-21

**Authors:** He Huang, Xiaojing Dong, Zuoyuan Du, Li Guo, Lan Chen, Xinming Wang, Simin Lu, Kun Yue, Zhihong Ren, Jianwei Wang, Lili Ren, Jianguo Xu

**Affiliations:** 1 NHC Key Laboratory of System Biology of Pathogens and Christophe Mérieux Laboratory, National Institute of Pathogen Biology, Chinese Academy of Medical Sciences & Peking Union Medical College, Beijing, China; 2 State Key Laboratory of Respiratory Health and Multimorbidity, Chinese Academy of Medical Sciences & Peking Union Medical College, Beijing, China; 3 National Key Laboratory of Intelligent Tracking and Forecasting for Infectious Diseases, National Institute for Communicable Disease Control and Prevention, Chinese Center for Disease Control and Prevention, Beijing, China; 4 Research Units of Discovery of Unknown Bacteria and Function (2018 RU010), Chinese Academy of Medical Sciences, Beijing, China; Victorian Infectious Diseases Reference Laboratory, Melbourne, Australia

**Keywords:** probiotics, *Lactobacillus*, SARS-CoV-2, inflammation, antiviral

## Abstract

Severe acute respiratory syndrome coronavirus 2 (SARS-CoV-2) targets epithelial cells in the respiratory tract, triggering an acute proinflammatory response and chronic lung inflammation. Probiotic supplementation has shown promise in reducing the nasopharyngeal SARS-CoV-2 viral load, diminishing symptom frequency and duration, and mitigating inflammation; thus, it is a potential strategy for treating coronavirus disease 2019 (COVID-19), which is caused by SARS-CoV-2 infection. In this study, we evaluated the effects of the oral administration of the *Lactobacillus plantarum* GUANKE strain, a gram-positive bacterium originally isolated from a healthy individual, on SARS-CoV-2 infection in a human ACE2 transgenic mouse model. We found that GUANKE significantly reduced inflammatory cell infiltration and pulmonary interstitial exudation in mice. The transcription of *CCL2*, *TNFA*, *IL1B*, *IL6,* and *IL17C* in the lungs was reduced. The protein levels of TNF-α, IL-1β, IL-6, and IL-17 in the lungs were significantly lower in GUANKE-treated mice than in control mice. The viral load in GUANKE-treated mice was lower than that in saline-treated mice, although this difference did not reach statistical significance. *L. plantarum* GUANKE can decrease SARS-CoV-2-induced lung inflammation in mice, suggesting its potential for use as an agent for treating SARS-CoV-2 infection.

**IMPORTANCE:**

Most otherwise healthy individuals develop only mild or moderate symptoms of coronavirus disease 2019 (COVID-19) caused by current strains of severe acute respiratory syndrome coronavirus 2 (SARS-CoV-2), and virus replication is mainly confined to the upper respiratory tract; however, the virus can infect the lower respiratory tract and promote inflammation. Probiotic supplementation has been shown to reduce nasopharyngeal SARS-CoV-2 viral load, reduce the overall number and duration of symptoms, and attenuate inflammation in clinical trials. We showed that a novel *L. plantarum* GUANKE strain alleviated SARS-CoV-2-induced pneumonia in mice. The transcription and production of inflammatory cytokines were suppressed, and GUANKE moderately reduced the viral load. *L. plantarum* GUANKE has the potential to become a candidate drug for the treatment of COVID-19 or other viral respiratory infections.

## INTRODUCTION

In December 2019, an emerging highly pathogenic human coronavirus, namely, severe acute respiratory syndrome coronavirus 2 (SARS-CoV-2), caused an outbreak of coronavirus disease 2019 (COVID-19), which was declared a pandemic by the World Health Organization (1). Four years after the emergence of SARS-CoV-2, new variants (e.g., JN.1) still circulate around the world and continue to be associated with increased hospitalizations (2). Thus, the ongoing exploration of innovative treatment approaches remains crucial in clinical practice.

Most otherwise healthy individuals develop only mild or moderate symptoms of COVID-19 caused by current strains of SARS-CoV-2, and virus replication is confined mainly to the upper respiratory tract; however, some people develop life-threatening pneumonia (3). SARS-CoV-2 can infect the lower respiratory tract and promote inflammation and coagulation, leading to pathological damage. Cytokine levels are strongly associated with disease severity and predict the progression of COVID-19 (4, 5). Anti-inflammatory therapy may help prevent further injury, but anti-inflammatory medications, such as corticosteroids, may delay the elimination of viruses and increase the risk of secondary infection (6). Probiotics are live microorganisms, typically bacteria or yeast, that not only support the health of the gut but also improve system function and regulation. The gut microbiome impacts systemic immune responses as well as local immune responses at distal mucosal sites, including the lungs (7–9). Probiotic supplementation reduced nasopharyngeal SARS-CoV-2 viral load, reduced the overall number and duration of symptoms, and attenuated inflammation in several clinical trials (10–13), indicating that it is a promising viable therapeutic and prophylactic option for treating COVID-19.

*Lactobacillus plantarum* is a gram-positive, facultatively anaerobic, rod-shaped bacterium that is distributed in the human intestinal tract. *L. plantarum* GUANKE (GUANKE) is a novel strain that was originally isolated from the fecal sample of a healthy individual. We found that GUANKE, as a probiotic, promoted SARS-CoV-2 vaccine-elicited immune responses by increasing interferon signaling and suppressing apoptotic and inflammatory pathways in mice (14). Moreover, GUANKE alleviated allergic rhinitis symptoms by modulating the functions of various cytokines and chemokines (15). In this study, we evaluated the effects of the oral administration of *L. plantarum* GUANKE on SARS-CoV-2 viral load and lung inflammation in a mouse model, and the findings may provide evidence for the use of *L. plantarum* GUANKE in the treatment of COVID-19.

## MATERIALS AND METHODS

### Ethics statement and animals

All the animal-related procedures were conducted according to the protocol approved by the Institutional Animal Care and Use Committee of the Institute of Laboratory Animal Science of the Chinese Academy of Medical Sciences, Beijing, China. Six- to eight-week-old male human ACE2 transgenic mice (C57BL/6-hACE2, T037630) were purchased from Gem Pharmatech and maintained under specific pathogen-free conditions with free access to water and mouse food.

### Bacterial culture

The *L. plantarum* GUANKE strain was routinely cultured in Man-Rogosa-Sharpe broth at 37°C in a CO_2_ incubator. Bacteria in the logarithmic phase of growth were washed with phosphate-buffered saline (PBS) containing 10% glycerol by centrifugation, and the bacterial sludge was preserved directly at −20°C for less than 5 days. Prior to use, the stored sludge was serially diluted with PBS, plated onto Man-Rogosa-Sharpe Agar, and incubated in a CO_2_ incubator for 24 h. The colonies were then counted to calculate the bacterial concentration (CFU/g). Before the oral inoculation of the mice, the bacterial sludge was resuspended in sterile PBS and diluted to the desired concentration.

### SARS-CoV-2 stock preparation

The IPBCAMS-WH-01/2019 strain of SARS-CoV-2 was isolated and identified by our laboratory (GISAID: no. EPI_ISL_402123) (1). The virus was cultured in Vero cells (ATCC, #CCL-81). The cells were cultured in Dulbecco’s modified Eagle’s medium (DMEM, Thermo Fisher Scientific) supplemented with 10% heat-inactivated fetal bovine serum (FBS) (HyClone), 100 U/mL penicillin, and 100 U/mL streptomycin at 37°C in a 5% CO_2_ humidified atmosphere. The cells were infected with SARS-CoV-2 at a multiplicity of infection of 0.5. The supernatant was collected at 48–72 h post-infection, clarified by centrifugation at 2,000 × *g* for 10 min, aliquoted, and stored at −80°C. The titer of the virus stock was determined via the 50% tissue culture infectivity dose (TCID_50_) method and calculated via the Reed and Muench method on Vero cells. The titer of the virus stock used in this study was 3.16 × 10^6^ TCID_50_/mL.

### SARS-CoV-2 infection

A nonlethal mouse model of SARS-CoV-2 infection was used in this study. The mice were anesthetized with 250 mg/kg of tribromoethanol and then inoculated with 10^5^ TCID_50_ of SARS-CoV-2 into both nostrils. The criteria that defined the successful establishment of the pneumonia model were as follows: broadening of alveolar septa; neutrophil, monocyte, and/or macrophage infiltration in the interstitial space and/or the alveolar space, with/without lung consolidation; the diffuse disruption of alveolar walls; apparent lung epithelial or endothelial cell death; and apparent hemorrhage in vessels, as observed through hematoxylin and eosin staining of lung sections at 3 days post-infection.

**FIG 1:**
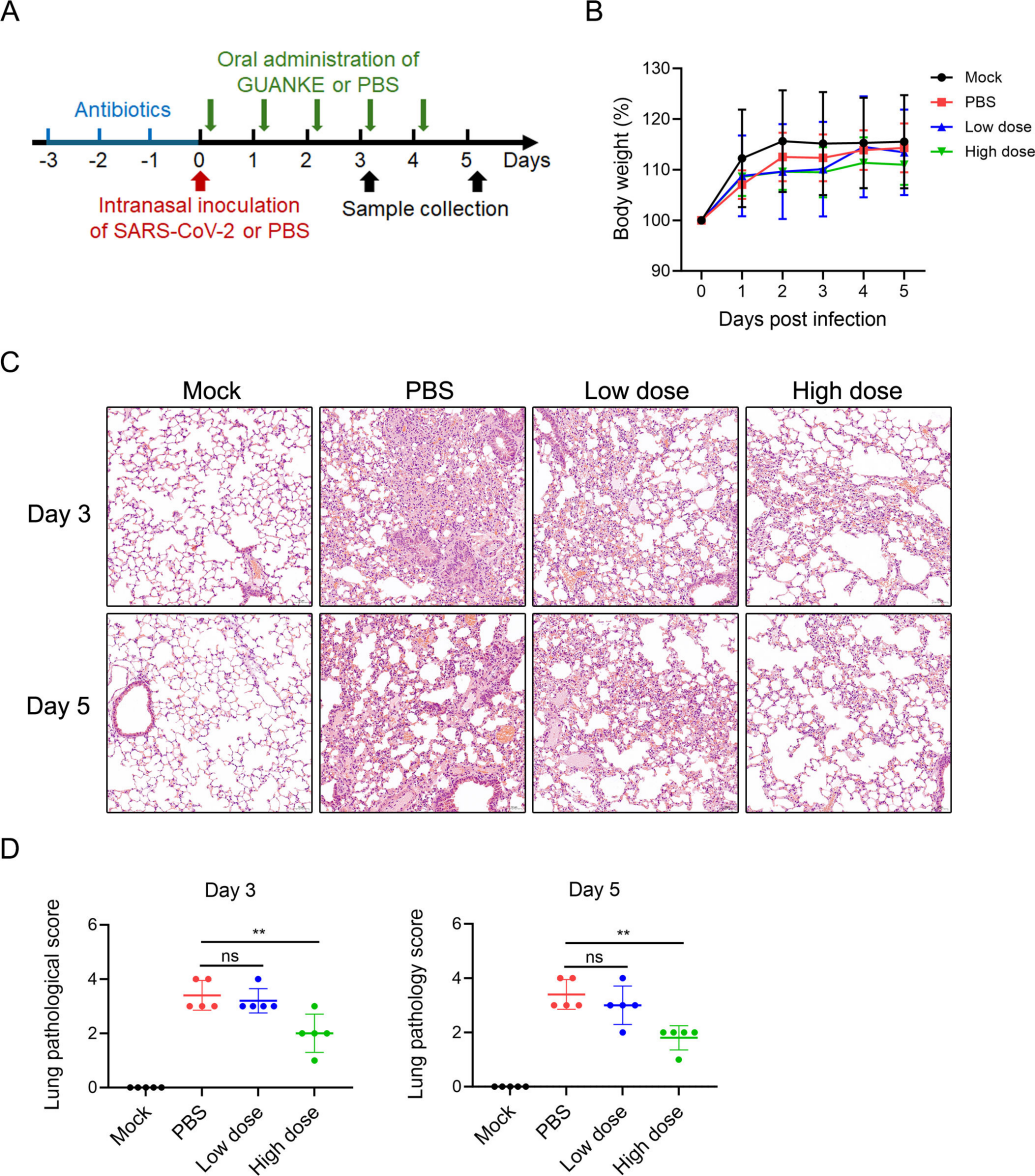
Oral administration of *L. plantarum* GUANKE reduced severe acute respiratory syndrome coronavirus 2 (SARS-CoV-2)-induced lung inflammation in C57BL/6-hACE2 mice. (**A**) Experimental design workflow. (**B**) Body weight was analyzed daily following SARS-CoV-2 infection (*n* = 5 in each group). (**C**) Hematoxylin-eosin staining of the left lungs was performed on the third or fifth days after SARS-CoV-2 infection. Representative images are shown (100×). (**D**) Pathological scores of the left lungs are provided. The data are presented as the means ± SDs. ns, not significant; **P < 0.01.

The animal experimental procedure is shown in Fig. 1A. Before the administration of *L. plantarum* GUANKE, the mice were given water supplemented with antibiotics (1 g/L ampicillin, 0.5 g/L vancomycin, 1 g/L gentamicin, and 1 g/L metronidazole) for 3 days to minimize the effects of the gut microbiome on the results. Then, the mice were intranasally inoculated with PBS (pH 7.4) or SARS-CoV-2. Given the virus stock titer of 3.16 × 10^6^ TCID_50_/mL, the inoculum volume was set at 32 μL. Then, 4 h after the inoculation, PBS or *L. plantarum* GUANKE was orally administrated. *L. plantarum* GUANKE was administered to each mouse daily for 5 days at either a low dose of 1.0 × 10^6^ CFU or a high dose of 1.0 × 10^10^ CFU. The body weights of the mice were collected daily after infection. The mice were euthanized, and tissue samples were collected on the third and fifth days after SARS-CoV-2 infection. The animal experiments were performed in a Biosafety Level 3 laboratory.

### Enzyme-linked immunosorbent assay (ELISA)

The right lung lobes of the mice were weighed and homogenized in 1 mL of cold PBS. The supernatant was collected from 900 µL of homogenate via high-speed centrifugation, and the concentration of cytokines was analyzed by ELISA. The remaining homogenates were stored for RNA extraction. ELISA kits for tumor necrosis factor-alpha (TNF-α, EM008-96), interleukin 6 (IL-6, EM004-96), monocyte chemoattractant protein-1 (MCP-1, EM018-96), interleukin 1-beta (IL-1β, EM001-96), interferon gamma (IFN-γ, EM007-96), and interleukin 17 (IL-17, EM015-96) were purchased from ExCell Bio. The assay was conducted following the manufacturer’s instructions.

### Quantification of viral titers

The supernatants mentioned above were diluted with DMEM to create 10-fold dilutions. Vero cells were seeded into 96-well plates the day before the experiment. Following a wash with DMEM, 100 µL of the serially diluted material was added to the cells (eight wells per dilution) and incubated at 37°C for 1 h. After another wash with DMEM, the cells were cultured in DMEM supplemented with 2% FBS for 3–5 days. The cell morphology was observed daily, and the endpoints on each row were documented with cytopathic effects. The TCID_50_ was calculated via the Reed–Muench method.

### Quantitative PCR (qPCR) analysis

Total RNA was extracted from 100 µL of the homogenate mentioned above via TRIzol reagent (Thermo Fisher Scientific) following the manufacturer’s instruction. Absolute quantification of SARS-CoV-2 RNA was performed via TaqMan Fast Virus 1-Step Master Mix (Thermo Fisher Scientific). Home-made standard samples were used for quantification. The primers and probes used in the reaction were as follows:

nCoV-F 5′-AACACAAGCTTTCGGCAGAC-3′
nCoV-R 5′-ACCTGTGTAGGTCAACCACG-3′
nCoV-P 5′-CAGCGCTTCAGCGTTCTTCGGAATGTCGC-3′

The expression of lung cytokines was analyzed via relative quantitative PCR. cDNA was synthesized using the High-Capacity cDNA Reverse Transcription Kit (Thermo Fisher Scientific). The reverse transcription products were amplified using SYBR Green PCR Master Mix (Thermo Fisher Scientific) following the manufacturer’s instructions, and the data were normalized to the level of Actin in each individual sample. The 2^−ΔΔCt^ method was used to calculate relative expression changes. The following primers were used:

mTNF-F 5′-CCCTCACACTCAGATCATCTTCT-3′
mTNF-R 5′-GCTACGACGTGGGCTACAG-3′
mIL6-F 5′-TAGTCCTTCCTACCCCAATTTCC-3′
mIL6-R 5′-TTGGTCCTTAGCCACTCCTTC-3′
mMCP1-F 5′-CTGAGTTGACTCCTACTGTGGA-3′
mMCP1-R 5′-TCTTCCCAGGGTCGATAAAGT-3′
mIL1B-F 5′-GAAATGCCACCTTTTGACAGTG-3′
mIL1B-R 5′-TGGATGCTCTCATCAGGACAG-3′
mIL17C-F 5′-CTCCTGCTTCTAGGCTGGTTG-3′
mIL17C-R 5′-CCACCTGGCACTTCGAGTTAG-3′
mIFNA2-F 5′-CTGCTGGCTGTGAGGAAATA-3′
mIFNA2-R 5′-GGCTCTCCAGACTTCTGCTC-3′
mIFNG-F 5′-ATGAACGCTACACACTGCATC-3′
mIFNG-R 5′-CCATCCTTTTGCCAGTTCCTC-3′
mACTB-F 5′-GGCTGTATTCCCCTCCATCG-3′
mACTB-R 5′-CCAGTTGGTAACAATGCCATGT-3′

### Histopathological examination

The left lungs of the mice were fixed by submersion in 15 mL of 10% phosphate-buffered formalin and incubation for 24 h, followed by embedding in paraffin. Then, 5-μm-thick sections were cut, stained with hematoxylin and eosin, and examined under a light microscope to evaluate histological changes. Pathological scores were obtained via the scoring system previously reported (16).

### Statistical analysis

All the data are presented as the means ± standard deviations (SDs). Statistical analyses were conducted via GraphPad Prism 10.0 software (GraphPad Inc.). Statistical significance was assessed via an unpaired two-tailed Student’s *t*-test for comparisons between two groups. In the figure legends, statistical significance is denoted as *P < 0.05 and **P < 0.01.

## RESULTS

### GUANKE reduced SARS-CoV-2-induced pneumonia

Since most patients infected with SARS-CoV-2 present mild to moderate symptoms, we used a nonlethal animal model of infection for this study. Despite the absence of weight loss in mice following SARS-CoV-2 infection, pathological examination of lung tissues revealed significant disruptions in the pulmonary alveolar structure, characterized by edema of the alveolar walls, thickening of the pulmonary interstitium, and notable infiltration of inflammatory cells (Fig. 1B and C). High-resolution images of the hematoxylin-eosin stained sections are shown in Fig. S1. In contrast, lung tissues from uninfected mice presented a normal structure with intact alveolar walls and no evidence of inflammatory cell infiltration in the alveolar cavity or pulmonary interstitium.

On the third and fifth days following the administration of GUANKE, pulmonary pathology revealed a significant reduction in inflammatory cell infiltration and pulmonary interstitial exudation in the high-dose group, leading to improved lung pathology but not in the low-dose group. The pathological score of the lung correlated with the pathological observations (Fig. 1D). These results indicate that oral administration of a high dose of GUANKE can reduce SARS-CoV-2-induced pneumonia in mice.

### GUANKE reduced the protein levels of inflammatory cytokines in the lungs

**FIG 2:**
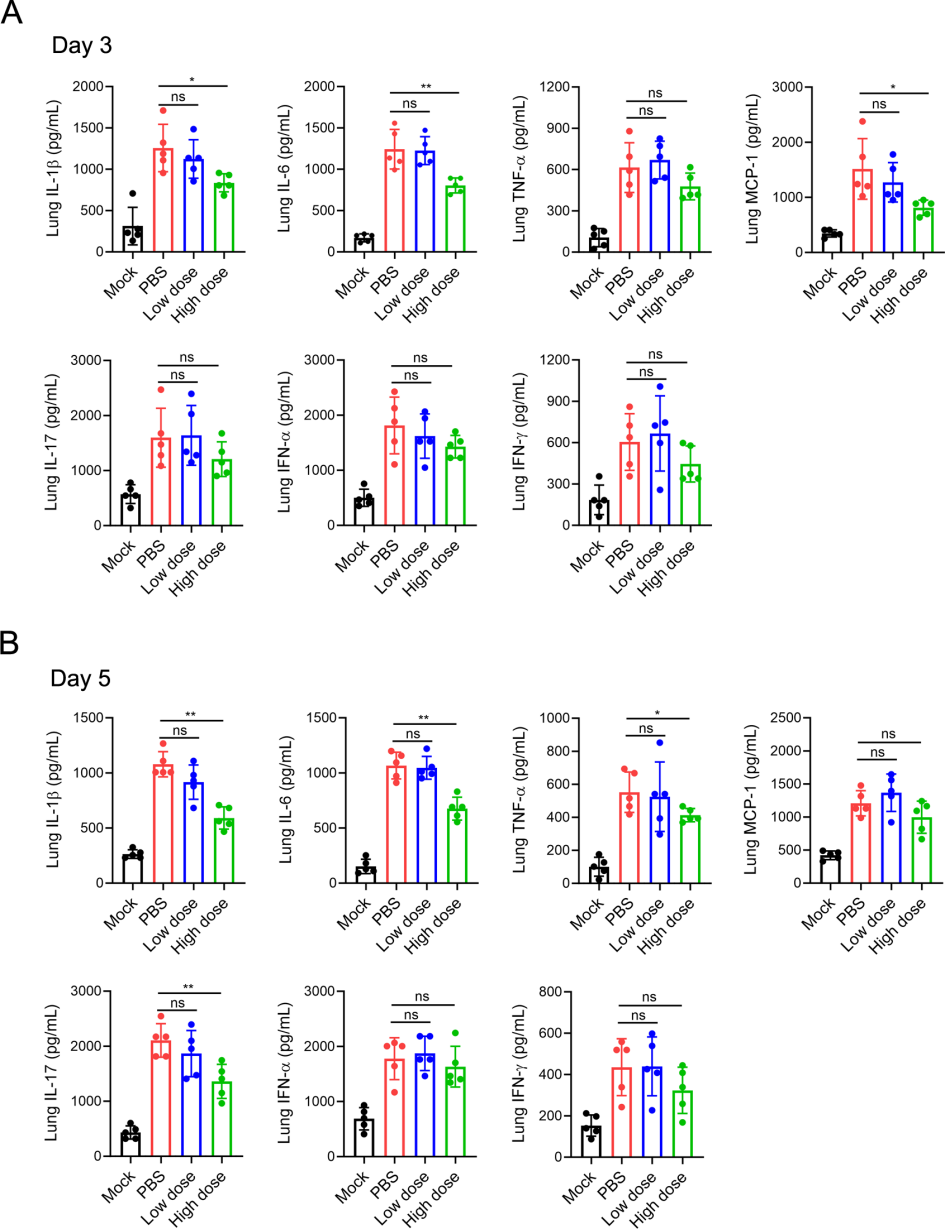
Oral administration of *L. plantarum* GUANKE reduced severe acute respiratory syndrome coronavirus 2 (SARS-CoV-2)-induced cytokine expression in the lungs. The right lung lobes of the mice were homogenized in phosphate-buffered saline (PBS) at 3 days (**A**) or 5 days (**B**) after SARS-CoV-2 infection. The concentrations of cytokines in the supernatants of the homogenates were analyzed via enzyme-linked immunosorbent assay (ELISA). The data are presented as the means ± SDs. ns, not significant; *P < 0.05; **P < 0.01.

We then analyzed whether the administration of GUANKE could inhibit the production of inflammatory cytokines in lung tissue by ELISA. As shown in Fig. 2A, on the third day after infection, SARS-CoV-2 infection induced a significant increase in inflammatory cytokines and a high dose of GUANKE significantly reduced the induction of MCP-1 and IL-6. On the fifth day after infection, a high dose of GUANKE reduced the induction of TNF-α, IL-1β, IL-6, and IL-17 (Fig. 2B). However, the protein levels of these cytokines did not significantly differ between the low-dose group and the PBS group on either the third or fifth day after infection.

### GUANKE downregulated the transcription of inflammatory cytokines in the lungs

**FIG 3:**
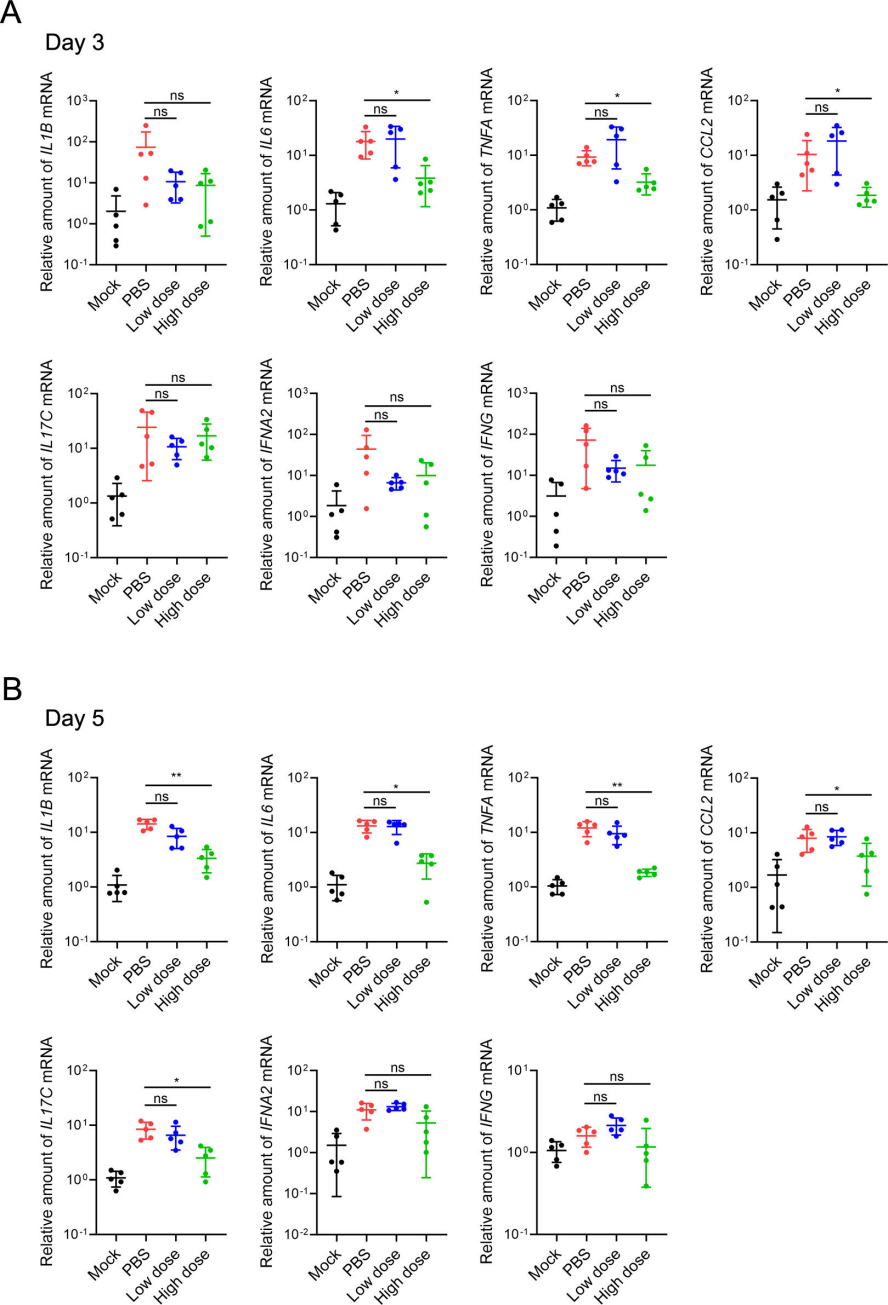
Oral administration of *L. plantarum* GUANKE reduced severe acute respiratory syndrome coronavirus 2 (SARS-CoV-2)-induced cytokine transcription in the lungs. The right lung lobes of the mice were homogenized in phosphate-buffered saline (PBS) at 3 days (**A**) or 5 days (**B**) after SARS-CoV-2 infection. Total RNA was extracted from the homogenates, and the transcription of cytokines was analyzed via relative quantitative PCR. The data are presented as the means ± SDs. ns, not significant; *P < 0.05; **P < 0.01.

The mRNA levels of these cytokines were also analyzed by real-time PCR. Similar to the protein level, the administration of a high dose of GUANKE reduced the transcription of *CCL2* (the gene encoding MCP-1), *TNFA,* and *IL6* on the third day after infection, and decreased the transcription of *CCL2*, *TNFA*, *IL1B*, *IL6,* and *IL17C* on the fifth day after infection (Fig. 3). Administration of a low dose of GUANKE did not alter the transcription of the cytokines on either the third or fifth day after infection. These results suggest that the inhibition of inflammatory cytokine production may contribute to the reduction in SARS-CoV-2-induced pneumonia caused by GUANKE administration.

### GUANKE-treated mice presented a moderate reduction in viral load

**FIG 4:**
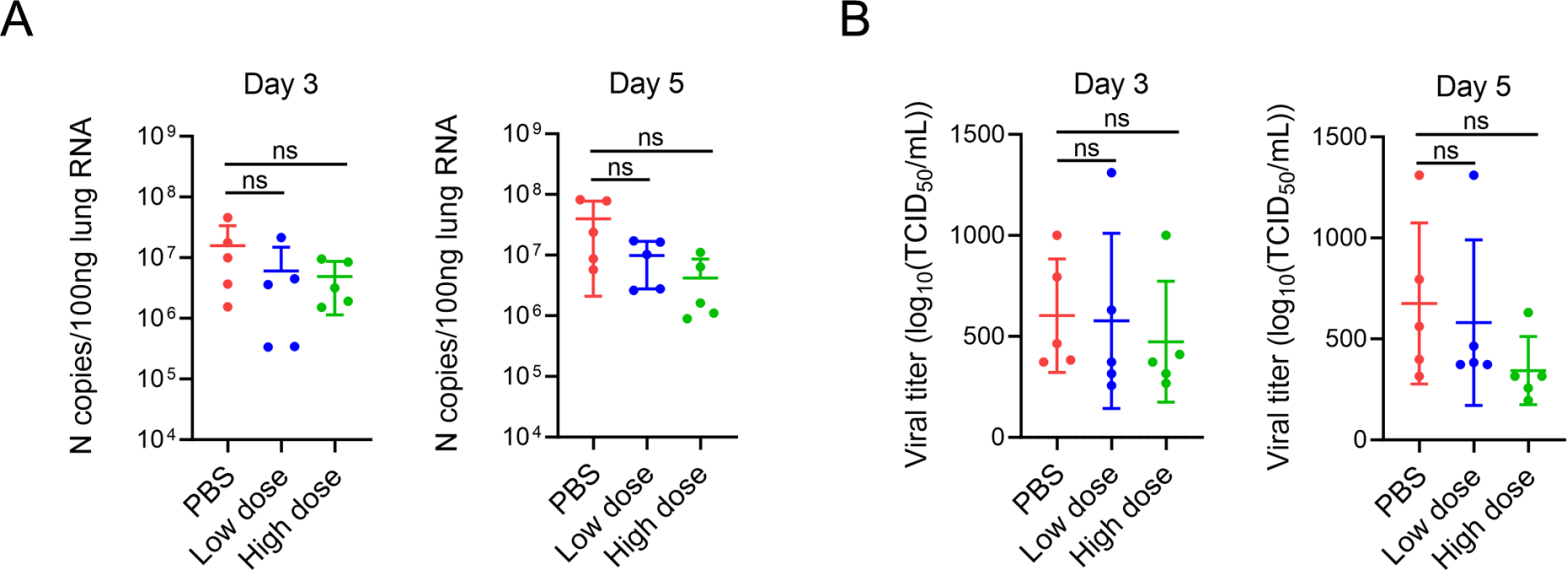
Oral administration of *L. plantarum* GUANKE had a limited effect on reducing the viral load in the lungs. (**A**) The right lung lobes of the mice were homogenized in phosphate-buffered saline (PBS) on the third or fifth day after severe acute respiratory syndrome coronavirus 2 (SARS-CoV-2) infection. Total RNA was extracted from the homogenates, and the copy number of the viral N gene was analyzed via quantitative PCR. (**B**) Viral titers in the supernatants of the homogenates were determined. The data are presented as the means ± SDs. ns, not significant.

Next, we analyzed the effects of GUANKE administration on viral replication in the lung. First, the viral titers in lung homogenates were determined. As shown in Fig. 4A, a high dose of GUANKE resulted in a reduction in the viral load in the lung on the third and fifth days after infection; however, this decrease was not statistically significant. No significant difference in viral load was observed between the PBS and the low-dose group. The expression of the N gene of the viral genome in the lung was also quantified via PCR (Fig. 4B). Similar to the viral titration results, the N gene copy number was reduced in the high-dose group, although the difference was not statistically significant. The administration of a low dose of GUANKE did not significantly alter the replication of the viral genome.

## DISCUSSION

Microbiome studies suggest that individuals with COVID-19 display a distinct intestinal microbiome dysbiosis that is closely correlated with disease pathophysiology (17, 18). Rebalancing the intestinal microbiome via probiotics may be effective for controlling COVID-19. Several studies have shown that probiotics and synbiotics have positive effects on patients with COVID-19, resulting in symptom alleviation, inflammation reduction, and notable decreases in mortality rates (19, 20).

*L. plantarum* is a gram-positive, facultatively anaerobic, rod-shaped bacterium that is distributed in the human intestinal tract. Its ability to reduce the risk of upper/lower respiratory tract infection and alleviate the respiratory symptoms of infected patients has been demonstrated (21, 22). The underlying immunological effects included enhancement of the phagocytic activity of granulocytes, reduction of the plasma proinflammatory cytokines IFN-γ and TNF-α, elevation of the anti-inflammatory cytokines IL-4 and IL-10, and activation of CD8^+^ T cells. However, the impact of oral *L. plantarum* administration on COVID-19 has not been elucidated. In this study, we found that the *L. plantarum* GUANKE strain alleviated SARS-CoV-2-induced pneumonia in mice. Oral administration of GUANKE significantly reduced the production of TNF-α, IL-1β, IL-6, and IL-17 induced by SARS-CoV-2 infection and reduced pathobiological damage in the lungs.

The beneficial metabolites secreted by probiotics termed postbiotics could promote immunomodulatory and antiviral effects (23, 24). Although our study did not investigate the detailed mechanisms by which oral probiotics regulate respiratory infections, it is believed that these probiotics exert systemic effects through the “gut–lung axis: by modulating mucosal immune function (25, 26).

Extract from *L. plantarum* Probio-88 strain significantly inhibited the replication of SARS-CoV-2 and the production of reactive oxygen species *in vitro* (27). It was postulated that the antiviral activity of *L. plantarum* Probio-88 was derived from the binding of plantaricin E and F to the viral helicase. Because GUANKE was orally administered in our study, direct antiviral effects might not have been observed. Neither the level of IFN-α nor the viral load significantly changed after GUANKE administration, likely because the immunomodulatory effects of GUANKE primarily regulate inflammatory responses rather than antiviral responses. Similarly, *L. plantarum* DR7 strain was found to alleviate the symptoms of upper respiratory tract infections by improving inflammatory parameters (22). However, further studies are needed to elucidate the underlying mechanisms by which oral *L. plantarum* regulates the lung inflammatory response.

The limitations of our study should also be noted. In our model, lung cytokine production peaked on day 3 post-infection (data not shown), and probiotics required several days to exhibit immunomodulatory effects (28). To better characterize the anti-inflammatory effects, we reduced the time between virus challenge and *L. plantarum* administration to 4 h. This timeframe is not consistent with clinical management in practice because there is an incubation period before symptoms appear, and diagnosis takes time. A more suitable animal model may be chosen to better simulate the typical infection timeline in the future. Moreover, the virus we used in the animal model was the prototype strain and circulating variants exhibit different behaviors in terms of pathogenesis and infectivity, making it difficult to directly translate our findings to real-world scenarios involving current variants.

In this study, we focused on the impact of GUANKE on acute infection with SARS-CoV-2. The post-COVID-19 condition (long COVID) occurs in patients after acute COVID-19 and is suggested to be caused by prolonged inflammation after recovery (29). The composition of the gut microbiome has also been reported to be associated with long COVID (30). Thus, supplementation of probiotics might also have positive effects on preventing long COVID.

In conclusion, our results demonstrated that *L. plantarum* GUANKE can alleviate SARS-CoV-2-induced lung inflammation by suppressing the transcription and production of inflammatory cytokines. *L. plantarum* GUANKE has the potential to become a candidate drug for the treatment of COVID-19 or other viral respiratory infections.

## Data Availability

The data underlying this article will be shared upon reasonable request to the corresponding author.

## SUPPLEMENTAL MATERIAL

Figure S1 spectrum.01603-24-s0001.pdf High-resolution images corresponding to Fig. 1C.

ASM does not own the copyrights to Supplemental Material that may be linked to, or accessed through, an article. The authors have granted ASM a non-exclusive, world-wide license to publish the Supplemental Material files. Please contact the corresponding author directly for reuse.
