# Supplementary figures and images for: Administration of Lactobacillus plantarum GUANKE alleviates SARS-CoV-2-induced pneumonia in mice
Source: Microbiol Spectr. 2024 Oct 21;12(12):e01603-24. doi: 10.1128/spectrum.01603-24 (PMC11619608; doi:10.1128/spectrum.01603-24)

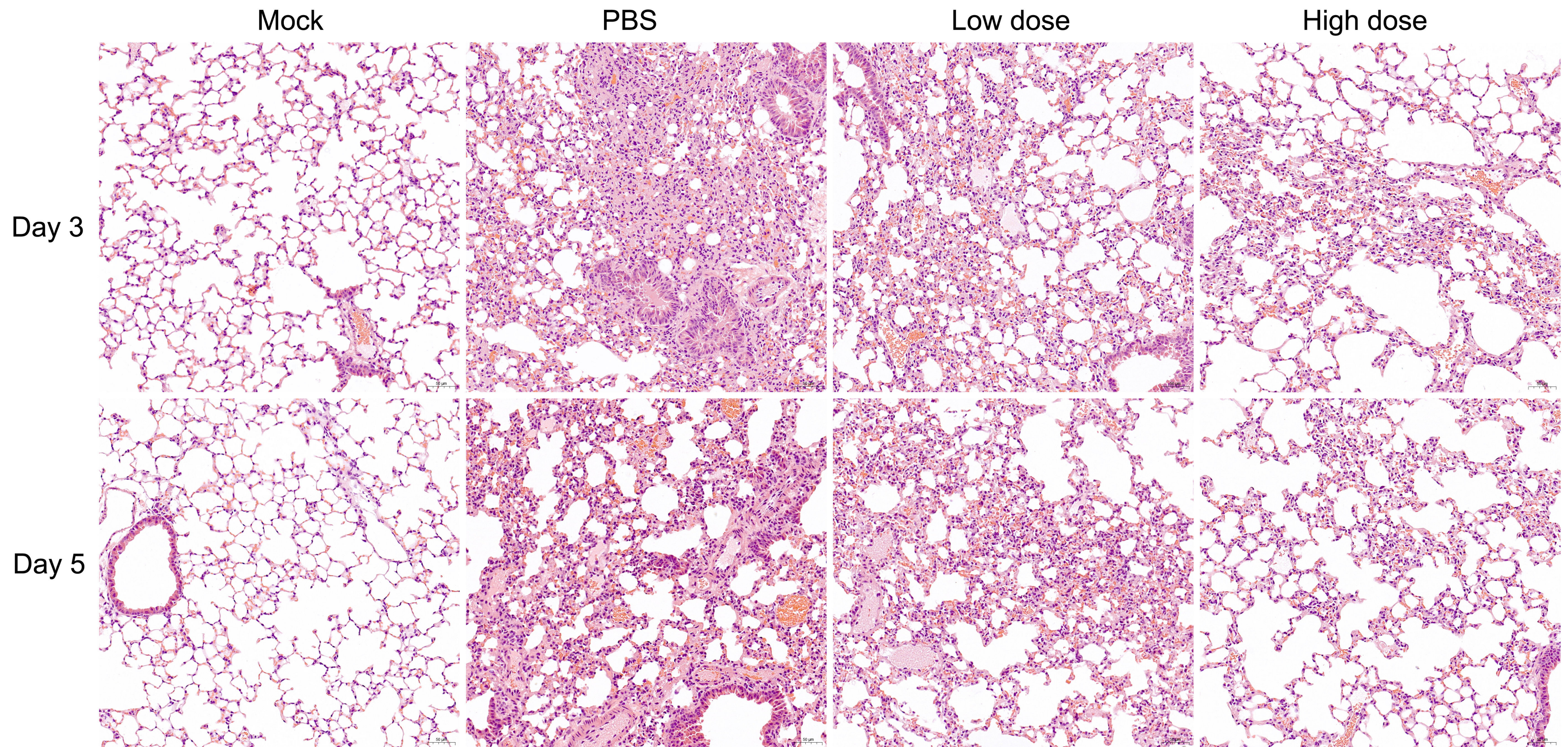

Figure S1. High-resolution images corresponding to Figure 1C.

Supplement: Figure S1 — High-resolution images corresponding to Fig. 1C. [file spectrum.01603-24-s0001.pdf]
